# Resilience to ghosting? A randomized controlled trial examining the consequences of ghosting on sleep quality in potential romantic relationships

**DOI:** 10.3389/fpsyg.2025.1742356

**Published:** 2026-01-27

**Authors:** Michael R. Langlais, Anabelen Pons, Olivia Dechert, Anna M. Murvich, Jeremy A. Bigalke

**Affiliations:** 1 Department of Human Sciences and Design, Baylor University, Waco, TX, United States; 2 Department of Health, Human Performance, and Recreation, Baylor University, Waco, TX, United States

**Keywords:** attraction, ghosting, mating, romantic relationships, sleep, sleep quality

## Abstract

**Introduction:**

Grounded in belongingness theory, the goal of this study is to understand the consequences of ghosting (an approach to ending relationships that involves ending all forms of communication with someone without explanation) on objective and subjective sleep quality in young adults seeking romantic relationships.

**Methods:**

Data for this experimental study comes from emerging adults recruited from a university in the Southern Central United States (*N* = 112) who were told that they would be put into a dating pool and matched with a romantic partner. All participants were randomly assigned to one of three conditions: ghosting (*N* = 40), courteous ending (*N* = 35), or a control condition (*N* = 37). In the ghosting condition, participants received a text message initiating conversation with a romantic partner “match” (laboratory confederate), but the conversation was abruptly ended after 2 hours. In the courteous ending group, participants conversed with a laboratory confederate, but after two hours the confederate politely ended the conversation and said that they would reach out to them in the future. The control condition received no text message. Participants wore Oura rings for two days and completed a sleep diary for two consecutive mornings (i.e., pre- and post-experimental manipulation) to capture objective and subjective sleep.

**Results:**

Contrary to our hypothesis, we did not observe any influence of acute experimental ghosting on objective or subjective sleep measures.

**Discussion:**

This study is unique in that it aimed to experimentally elicit relationship stress using a novel low-investment ghosting protocol to assess the influence of relationship stress on objective and subjective sleep parameters. In addition, the study demonstrates the feasibility and limitations of a controlled experimental protocol for modeling ghosting in early-stage romantic contexts, providing a foundation for future experimental research on relational disengagement.

## Introduction

The manner in which people choose to end relationships has evolved, with a growing number opting for a phenomenon known as ghosting. Ghosting is described as a dissolution strategy in which one partner abruptly ceases all communication to terminate the relationship (LeFebvre et al., 2019; LeFebvre and Fan, 2020). Initial studies have shown that ghosting can have negative impacts on relational health (LeFebvre and Carmack, 2020; Navarro et al., 2020). Additionally, Langlais et al. (2024) found that ghosting is associated with elevated heart rate and blood pressure. However, few studies have examined the impact of ghosting for sleep quality. Sleep quality is important because it is directly correlated to mental and physical health (Luyster et al., 2012; Yap et al., 2020). Therefore, the goal of this study is to understand the consequences of a low-investment ghosting experience (i.e., conversations with a potential partner for a few hours that end abruptly) for objective and subjective sleep quality in romantic relationships.

This study offers several important contributions. First, it extends the growing body of research on ghosting by examining the impact of low-investment ghosting beyond emotional and relational outcomes, addressing a notable gap in the literature related to physical health - specifically, sleep quality. Low investment ghosting refers to minimal investment relationship situations, where participants talk with a potential romantic partner for a few hours before communication is abruptly ceased without explanation. Since both objective and subjective sleep are strongly linked to overall well-being (Kohyama, 2021; Luyster et al., 2012), particularly for emerging adults (Vail-Smith et al., 2009), understanding how relational disruptions like ghosting affect sleep can help practitioners, educators, and lay audiences. Second, this study extends the literature by using an experimental manipulation to potentially evoke low-investment ghosting stress. By exploring physiological and experiential dimensions of sleep, this study can offer insight into how relational stress manifests in the body, potentially identifying ghosting as a risk factor for sleep disturbances. Essentially, this study examines whether minimal investment ghosting affects sleep. Beyond testing hypothesized effects on sleep, the present study also contributes by evaluating an experimental approach to simulating minimal investment ghosting in a controlled and ethically appropriate manner, thereby helping to delineate boundary conditions under which ghosting-related effects may emerge.

## Literature review

Sleep is a critically important process with beneficial effects on both psychological and physiological health outcomes (Luyster et al., 2012). Conversely, sleep deprivation, restriction, and disruption are associated with adverse health outcomes. One factor which might significantly impact sleep quality is stress exposure (Yap et al., 2020; Yap et al., 2021; Yoo et al., 2023). Numerous studies have shown that daily fluctuations in stress exposure are associated with decrements in both subjective and objective sleep parameters (Åkerstedt et al., 2012; Yap et al., 2020; Yap et al., 2021; Yoo et al., 2023). Furthermore, poor sleep is closely linked to increased rates of depression among college students, suggesting that sleep disturbances may contribute to emotional instability and reduced academic success (Dinis and Bragança, 2018; Jalali et al., 2020).

However, while previous studies have assessed the influence of general, nonspecific stress exposure on temporal sleep patterns, fewer have assessed the influence of relationship stress specifically on sleep parameters (Xie and Feeney, 2024; Yap et al., 2020; Yap et al., 2021; Yoo et al., 2023). There are numerous relationship-specific mechanisms which might facilitate reductions in sleep quality including relationship conflict, negative partner interactions, among others (Xie and Feeney, 2024). Indeed, prior research has indicated a similar negative influence for relationship stress on sleep efficiency and disruption as that observed in response to general, nonspecific stress (Hasler and Troxel, 2010; Haydon and Salvatore, 2023). Recent work from Langlais et al. (2025) has further shown that the influence of acute relationship stress on sleep may be influenced by trait measures of relationship stress, suggesting the interacting influence of chronic and acute relationship stress on sleep outcomes. Despite these advances in the literature, few studies have assessed how relationship stressors specific to current romantic relationship culture impact young adult sleep health.

A recent relationship stressor receiving attention in the literature is ghosting. Ghosting is a dissolution strategy in which one partner abruptly ceases all communication, both online and offline, to end a romantic relationship without explanation (LeFebvre et al., 2019; Koessler et al., 2019). This relationship dissolution tactic involves behaviors such as ignoring messages, calls, and texts, unfollowing or blocking someone on social media platforms like Instagram or Snapchat, and ceasing any other forms of communication (Freedman et al., 2019). Schokkenbroek et al. (2025) provided a theoretical analysis of ghosting, emphasizing three key pillars that define the behavior. The first pillar, relationship type, considers the kinds of connections in which ghosting occurs, including romantic, platonic, and professional relationships. The second pillar, technology context, examines the role of digital communication in facilitating ghosting, while the third pillar, timing, addresses whether the cessation of contact is sudden or gradual.

Ghosting is a common experience in modern dating culture. Research indicates that the majority of young adults have either ghosted someone or been ghosted themselves (LeFebvre et al., 2019). While exact prevalence rates vary, ghosting is particularly frequent among young adults who often engage in short-term or exploratory romantic relationships. Recent research has started to focus on the health consequences of ghosting in romantic relationships. Although ghosting appears to be an easy way to terminate a relationship, it could be as detrimental as an official romantic breakup. There are similarities between a breakup that occurs through a conversation versus those that have no conversation (i.e., ghosting), including declines in self-esteem and increase in stress, anxiety, and depressive symptoms (Field et al., 2021; Langlais et al., 2024; Murray et al., 2002). Ghosting essentially prevents individuals from expressing their emotions and being heard, which are critical for maintenance of self-esteem (Vilhauer, 2015). Freedman and Powell (2024) found that ghosting is a common strategy for ending relationships, often used to avoid conflict or difficult conversations. While it occurs across romantic and platonic contexts, it is generally viewed negatively and can cause significant emotional distress for the person being ghosted. The study also highlighted that individual differences, such as attachment style and beliefs about relationships, influence both the likelihood of ghosting and how it is experienced. Additionally, ghosting can prompt physiological changes. For instance, in an experimental study by Langlais et al. (2024), participants exposed to a ghosting manipulation demonstrated short-term increases in heart rate and blood pressure. However, several limitations temper the extent to which these physiological responses can be linked to downstream outcomes such as sleep quality. The study had a relatively small sample size and may have been underpowered, several effects were only marginally significant (*p* < 0.10), and the physiological changes emerged during a brief 15-min interaction and began returning toward baseline by the end of the session. Thus, although these findings suggest that ghosting can elicit acute physiological arousal, they provide limited evidence regarding whether such short-lived responses translate into longer-term outcomes like sleep quality.

## The present study

This study is grounded in belongingness theory (Baumeister and Leary, 1995), which states that humans have a fundamental need to form and maintain interpersonal relationships. By having this need met, individuals would experience benefits in their psychological and physiological health. Given what we know about ghosting, the abrupt ceasing of meaningful conversation likely threatens individuals’ need to belong, thus hindering physiological health. We have an intrinsic need to connect with others, particularly those we are romantically interested in, and when that need is threatened, it is possible that individuals will lose sleep and experience lower sleep quality. Given this information, we *hypothesize that experiencing ghosting will cause changes in both subjective and objective sleep parameters, compared to experiencing the end of a conversation or not being ghosted.* The present study tests a lower-bound, early-stage form of ghosting that occurs prior to relationship establishment, allowing for an experimental examination of whether even minimal, acute experiences of relational disengagement are sufficient to disrupt objective or subjective sleep.

## Methods

### Ethical considerations

All participants provided knowledgeable consent to participate in the present study. All participants were also debriefed after completion of the study and informed of all deception employed throughout the experimental protocol. The present study was approved by the University of Baylor Institutional Review Board.

### Participants

Participants for this study were undergraduate college students who were 18 years and older, romantically single, and enrolled at the university where the study was being conducted. A total of 131 students expressed interest in participating. However, 11 participants never responded to the research team’s request to schedule a meeting, four participants did not show up for their orientation meeting, and four did not pass the manipulation check. Therefore, 112 individuals participated in the remainder of the study. Most participants identified as female (71.4%) and the rest identified as male (28.6%). The average age of participants was 19.95 (*SD* = 1.30; Range 18 to 25). Most participants listed their ethnicity as White/Caucasian (68.8%), followed by Hispanic (16.1%), Asian or Pacific Islander (8.0%), Black/African American (3.6%) or other (3.6%). The majority of participants identified as heterosexual (95.5%), followed by bisexual (2.7%), homosexual (0.9%), or preferred not to say (0.9%). Participants’ year in college were as follows: 22.3% first year, 25% second year, 25% third year, 25% fourth year, and 2.7% fifth year or more. One participant was diagnosed with sleep apnea (0.8%) and two participants were diagnosed with other sleep disorders (1.7%). Inclusion of these participants did not alter the primary findings of the study and therefore were retained to ensure statistical power.

### Procedures

Potential participants in this study were recruited at a midsize university in the southern central United States. Flyers were dispersed around campus, and information was shared with students via course management software. Recruitment flyers described a study that required college students who were 18 and older and romantically single to sign up to participate in a “match-making” study designed to help participants find a romantic partner. However, the goal of the study was to understand the impact of ghosting on subjective and objective sleep quality, which was not made known to participants until after the study was completed.

Those who were interested in participating were scheduled for an in-person orientation session with laboratory staff. These meetings were held in communal lab space. When an orientation meeting was scheduled (which we refer to as D1, meaning their first day in the study), participants were sent the day and time of the meeting, the location of the meeting, and a copy of the informed consent. The first part of this meeting was used to review the consent form and answer any participant questions. Once the participants signed the consent, they were fitted for and provided an Oura ring for the duration of this study, which was used to track objective sleep (Altini and Kinnunen, 2021). Participants were given information regarding how to use the Oura ring and the Oura application. Specifically, participants were instructed to wear the ring at all times except when lifting weights so as to not damage the ring. Importantly, participants were blinded to their data in the Oura mobile app through specialized research access granted to the researchers via Oura teams. Participants were then instructed to come back to the lab for two consecutive days (which we refer to as D2 for the second time point in the study and D3 for the following day).

On D2, participants received an online morning survey with questions regarding subjective sleep using a validated subjective sleep diary (Carney et al., 2012). Next, participants met with the Principal Investigator in his office between 8 a.m. and 2 p.m., where they first completed an online survey regarding demographic information, such as age, ethnicity, and education. They also answered questions regarding whether they had any sleep disorders or psychiatric conditions. Participants also answered 10–12 open-ended questions regarding their interests and hobbies to provide the guise of a match-making service. Participants were also instructed to upload a picture of themselves and provide their phone number at the end of the survey.

Once participants completed the online survey, using information regarding the participants’ sexual orientation provided in this online survey, participants were given pictures of 20–30 ‘potential partners,’ who participants were told that these persons were also enrolled in this match-making service. These pictures were of individuals who looked to be between 18 and 25 to mirror the age of the participants in this study found using the free and public databases *pixabay* and *unsplash*. The research team selected the pictures together to ensure that a diversity of potential partners was presented to participants. Participants were told to select 1 or 2 pictures of the individuals they found the most attractive. Once participants selected these pictures, the Principal Investigator pretended to enter information onto a spreadsheet. For participants in the control group, the Principal Investigator scheduled a time for the participant to come back the next day to return their ring once data was collected from the Oura ring. Participants in the control group were not told that they would receive any messages from potential partners. For the rest of the participants, the Principal Investigator said that the potential partner the participant selected was coming back to the lab later that day to return their ring, and that the participant’s phone number that they provided on the online survey would be shared with the potential partner in order to determine if there was a match. Next, the Principal Investigator scheduled a time for the participant to return their Oura ring the following day. This activity was the last part of the D2 meeting.

At the conclusion of the D2 meeting, the Principal Investigator randomly placed the participant into one of three conditions: (1) a ghosting condition, in which a research assistant posing as the potential partner texted with the participant for 2 h before ghosting them (i.e., abruptly ending the conversation without warning); (2) a courteous ending condition, in which the research assistant posing as a potential partner texted with the participant for 2 h, but ended the conversation, saying, “It was great talking with you but I have a lot of school work to do. Maybe we can talk again soon”; and (3) a control group, in which the participant did not receive any messages. Including a courteous ending condition allows the study to distinguish the effects of abrupt, unexplained rejection (ghosting) from a socially normative, polite termination of interaction. This comparison helps isolate the psychological impact of ambiguity and lack of closure, which are thought to be central to the negative outcomes associated with ghosting. By providing a condition in which the conversation ends respectfully and with a reason, the study can assess whether negative emotional responses are driven primarily by the abruptness and unpredictability of ghosting rather than the end of the interaction itself.

All messages were sent between the hours of 3 to 7 p.m.; once a participant responded, conversations lasted approximately 90–120 min, although the frequency in response rates varied, the average number of messages sent between participants and research assistants was 22.45 (*SD* = 12.33). Three participants said they never received messages from the research team and were moved to the control group (they may have entered their phone number incorrectly in the survey, or the research team may have typed the number incorrectly when sending a text message). The two-hour period was chosen to provide participants with enough time to establish a meaningful, interactive conversation while maintaining ecological validity consistent with typical adolescent texting behaviors. Additionally, a 90–120-min conversation window was selected to balance empirical precedent with practical considerations for participant well-being. Prior work using extended messaging interactions has demonstrated that longer exchanges can promote a sense of relational connection and conversational investment (e.g., Luo and Tuney, 2015). More recently, van der Zanden and Schokkenbroek (2024) showed that sustained online interactions can meaningfully shape individuals’ perceptions of relational dynamics, including experiences of rejection, suggesting that extended messaging provides a context in which interpersonal processes can unfold. Building on this evidence, we anticipated that a 90–120-min exchange would be sufficiently engaging for the manipulation to have a measurable impact. At the same time, this duration aligns with institutional review board recommendations to avoid excessively long interactions that could unduly intensify potential negative emotional or physiological consequences of the ghosting manipulation. Thus, the selected time frame reflects both empirical support for meaningful engagement in extended online conversations and ethical guidance aimed at minimizing participant burden and risk.

An *a priori* power analysis was conducted in G*Power using a repeated-measures ANOVA (within–between interaction) to reflect the structure of the study design. Parameters were set to a medium effect size (*f* = 0.25), *α* = 0.05, and power = 0.80. Following conventions for repeated-measures designs, we specified a moderate correlation among repeated measures (r = 0.50) and used the default nonsphericity correction (*ε* = 1.0). Under these conditions, G*Power indicated that approximately 90 participants (30 for each group) were required; the final sample (*N* = 116) exceeded this threshold. We selected a medium effect size based on general conventions in social and behavioral research when prior empirical estimates are unavailable. To our knowledge, no prior studies have reported effect sizes for the impact of ghosting on sleep quality specifically, and thus no field-based estimate was available to guide a more precise calculation. We acknowledge that if the true effect is smaller (e.g., *f* = 0.10), the present study would have been underpowered to detect it.

None of the content of the messages were saved to maintain confidentiality. To ensure that the condition that participants were in did not dictate the flow of conversations between the research assistant and the participant, the research assistants were not told what condition the participant was in until 10 min before their two-hour period of texting was scheduled to end. For the current study, research assistants did not use their own phones, but used phones provided by the university in which data collection occurred. The research team bought SIM cards to use for the current study to minimize the ability of participants finding out that they were texting someone who was posing as a potential partner. The research assistants were given instructions about how to initiate conversations and engage in healthy and safe dialogue with a potential partner (for instance, they were told not to give away any personal information or engage in any sexual dialogue); no issues of this nature arose during the course of this investigation (some participants asked the research assistants for their social media accounts, but the research assistants were told to tell the participants that they were taking a break from social media to help minimize any searching of potential partners).

The morning of D3, participants completed the sleep diary again in reference to how they slept the previous night (i.e., D2 night), which was emailed to them at 6 a.m. Participants were directed to complete the survey as soon as possible upon awakening. Next, participants met with the Principal Investigator, in which the participant was told the true goal of the study. During this time, the Principal Investigator engaged in a manipulation check to determine if the participants believed they were talking to an actual participant; three participants in the ghosting condition and one in the courteous ending condition stated that they questioned the validity of the person they were talking with. These participants were removed from the final analysis. Participants were also asked about their well-being after being told this information, and no participants indicated any psychological distress by participating (information for free counseling services on and off campus were provided in the consent form and during this debriefing period). Participants also returned their Oura rings during this meeting and were compensated for their participation, concluding the study.

## Measures

Table 1 displays means, standard deviations, and correlations across all study variables.

**Table 1:** Means, standard deviations, and correlations of study variables.

Variable	Mean	SD	1	2	3	4	5	6	7	8	9	10	11	12	13	14	15	16	17	18	19	20	21	22
1. OBJ - Day 2: Total sleep time (min)	406.83	77.63	–	−0.05	0.26**	0.40**	−0.15	0.05	0.84**	0.12	0.06	0.28**	0.15	0.22*	−0.03	0.17	0.05	−0.02	0.03	0.27**	0.16	0.06	0.13	0.00
2. OBJ - Day 2: Sleep efficiency (%)	89.88	4.23		–	−0.39**	−0.91**	−0.19*	0.18	−0.06	−0.05	−0.47**	−0.19*	0.08	−0.11	0.36**	−0.30**	−0.38**	−0.17	0.13	−0.05	−0.01	−0.08	−0.03	0.06
3. OBJ - Day 2: Sleep onset latency (min)	15.28	12.84			–	0.44**	−0.18	−0.04	0.14	0.27**	0.13	0.01	−0.05	0.06	−0.23*	0.30**	0.21*	0.01	0.00	0.01	0.11	0.06	0.04	−0.05
4. OBJ - Day 2: Wake after sleep onset (min)	47.17	24.79				–	0.14	−0.14	0.34**	0.12	0.44**	0.24*	0.00	0.16	−0.35**	0.35**	0.36**	0.13	−0.09	0.11	0.07	0.06	0.06	−0.04
5. OBJ - Day 2: Heart rate (bpm)	63.27	9.49					–	−0.52**	−0.11	−0.06	0.09	0.03	0.05	−0.12	−0.01	0.01	0.00	0.79**	−0.51**	−0.05	0.00	−0.01	0.02	0.05
6. OBJ - Day 2: Heart rate variability (ms)	64.61	38.23						–	0.04	0.08	−0.16	−0.13	−0.04	0.22*	0.14	0.06	−0.05	−0.53**	0.89**	0.09	−0.04	−0.11	−0.07	−0.08
7. SUBJ - Day 2: Total sleep time (min)	427.14	77.79							–	0.03	0.02	0.28**	0.18*	0.15	−0.03	0.12	0.07	0.01	0.02	0.32**	−0.04	0.03	0.02	0.05
8. SUBJ - Day 2: Sleep onset latency (min)	19.54	19.35								–	0.02	0.19*	−0.28**	0.23*	−0.13	0.19*	0.19*	0.00	0.11	0.09	0.45**	0.06	0.13	−0.21*
9. SUBJ - Day 2: Wake after sleep onset (min)	5.12	12.66									–	0.37**	−0.14	0.13	−0.05	0.13	0.08	0.05	−0.16	0.16	0.01	0.13	0.07	0.06
10. SUBJ - Day 2: Awakenings (#)	1.33	1.35										–	−0.31**	0.19*	−0.19*	0.10	0.21*	0.15	−0.19	0.15	0.00	0.27**	0.31**	0.01
11. SUBJ - Day 2: Sleep quality (a.u.)	3.57	0.81											–	−0.13	0.21*	−0.20*	−0.24*	−0.01	0.01	−0.09	−0.22*	−0.13	−0.07	0.40**
12. OBJ - Day 3: Total sleep time (min)	419.02	70.24												–	−0.07	0.24*	0.43**	−0.10	0.18	0.76**	0.13	0.11	0.26**	0.02
13. OBJ - Day 3: Sleep efficiency (%)	89.95	3.43													–	−0.36**	−0.90**	−0.17	0.17	−0.05	−0.15	−0.25**	−0.10	0.24*
14. OBJ - Day 3: Sleep onset latency (min)	15.48	12.49														–	0.43**	0.03	0.05	0.26**	0.23*	−0.06	−0.05	−0.01
15. OBJ - Day 3: Wake after sleep onset (min)	48.09	20.12															–	0.14	−0.09	0.32**	0.19	0.26**	0.20*	−0.23*
16. OBJ - Day 3: Heart rate (bpm)	63.88	8.52																–	−0.66**	−0.01	0.00	0.01	0.02	0.02
17. OBJ - Day 3: Heart rate variability (ms)	66.25	37.42																	–	0.00	0.05	−0.12	−0.08	−0.10
18. SUBJ - Day 3: Total sleep time (min)	433.81	75.45																		–	−0.09	0.07	0.10	0.19*
19. SUBJ - Day 3: Sleep onset latency (min)	18.78	23.43																			–	0.05	0.20*	−0.34**
20. SUBJ - Day 3: Wake after sleep onset (min)	5.10	10.29																				–	0.45**	−0.23*
21. SUBJ - Day 3: Awakenings (#)	1.32	1.50																					–	−0.32**
22. SUBJ - Day 3: Sleep quality (a.u.)	3.73	0.86																						–

BPM = beats/min; min = minute; ms = milliseconds; a.u. = arbitrary units.

*p < 0.05; **p < 0.01.

### Objective sleep quality

Sleep was objectively monitored throughout the study using Oura rings (Ōura Health, Oulu, Finland), commercially available wearable devices designed for noninvasive sleep tracking in home environments with minimal participant burden. Participants were instructed to wear the rings continuously for the duration of the study. Sleep detection relied on the Oura ring’s proprietary multi-sensor algorithm, which has shown high validity in prior research. Specifically, validation against gold-standard polysomnography demonstrated sensitivity and specificity ranging from 72 to 98% for detecting sleep and wake periods (Altini and Kinnunen, 2021). A recent evaluation of the same generation of Oura ring used in this study found an epoch-by-epoch sensitivity of 95% and specificity of 73% when compared to multi-night ambulatory polysomnography (Svensson et al., 2024). The present study employed the Oura Sleep Staging Algorithm 2.0 to analyze sleep data. Objective sleep measures included total sleep time (TST_obj_; total minutes asleep), sleep efficiency (SE_obj_; percentage of time in bed spent asleep), wake after sleep onset (WASO_obj_; minutes awake after falling asleep), and sleep onset latency (SOL_obj_; time taken to fall asleep). To reduce potential bias, participants were blinded to their sleep data and did not have access to nightly sleep summaries, thereby minimizing reactivity or changes in sleep behavior due to monitoring.

### Subjective sleep quality

Participants reported their subjective sleep on the mornings of D2 and D3 using an established sleep diary (Carney et al., 2012). Participants were asked each morning to estimate their nightly, subjective total sleep time (TST_subj_), sleep onset latency (SOL_subj_), number of awakenings (Awake_subj_), wake after sleep onset (WASO_subj_), and perceived quality of sleep (QOS_subj_) using a 1 (*very poor*) – 5 (*very good*) Likert scale.

### Nocturnal heart rate and heart rate variability

Nocturnal heart rate and heart rate variability are often used to estimate autonomic (specifically parasympathetic) regulation at night (Bigalke et al., 2023; Kerkering et al., 2022). Oura rings continuously tracked nocturnal heart rate and heart rate variability (SDNN) every 5-min throughout the night. Average nightly nocturnal heart rate and heart rate variability are presented throughout the results. Heart rate and heart rate variability collected from the Oura ring show high levels of agreement when compared to gold-standard electrocardiogram assessment (Kinnunen et al., 2020).

### Ghosting experimental manipulation

Participants texted with a research assistant posing as a potential partner for 2 h. Rather than ending the conversation, the research assistant was instructed to cease texting the participant. The participant could continue texting the research assistant, but the research assistant was told not to respond. This condition was compared to a control condition, in which participants did not receive a text message, and the courteous ending condition, in which participants were told that they were unable to continue texting that day in order to study.

## Data analysis

Statistics were performed using commercially available software (SPSS statistics). Participant characteristics were quantified using descriptive statistics, and group differences were assessed using one-way ANOVA or chi-square analyses where appropriate. Additionally, 3 (experimental condition) x 2 (day/night 2 and day/night 3) repeated measures ANOVAs were conducted to capture main effects of experimental condition, time, and their interaction on key outcome objective and subjective sleep variables of interest. Bonferroni post-hoc analyses were conducted to capture significant differences across conditions. *p*-values presented from Bonferroni comparisons are the product of unadjusted *p*-values and the number of comparisons being made, as performed by the statistical software utilized (SPSS). Therefore, a significance level of *α* < 0.05 was used for all statistical tests. All values presented are mean (SD) unless otherwise denoted. In addition to test statistics and *p*-values, effect sizes are reported for all comparisons to facilitate interpretation and comparability across studies. Cohen’s *d* is reported for pairwise contrasts, and partial η^2^ is reported for omnibus tests.

## Results

Participant characteristics and demographics are shown in Table 2. There were no significant differences in any demographic variables between groups, indicating that groups were well-matched.

**Table 2:** Participant characteristics (*N* = 112).

Variables	Ghosting (*N* = 40)	Courteous ending (*N* = 35)	Control (*N* = 37)	F/χ^2^
Sex (M/F)	15/25	8/27	9/28	2.45
Age (years)	19.77 (1.29)	20.11 (1.23)	19.97 (1.38)	0.64
Race/Ethnicity				
White/Caucasian (#)	28 (70.0)	26 (74.3)	23 (59.0)	7.13
Black/African American (#)	0 (0.0)	2 (5.7)	2 (5.2)	
Asian/Pacific Islander (#)	2 (5.0)	2 (5.7)	5 (12.8)	
Hispanic (#)	8 (20.0)	5 (14.3)	5 (12.8)	
Other (#)	2 (5.0)	0 (0.0)	2 (5.2)	
Sexuality				
Heterosexual (#)	38 (95.0)	34 (97.1)	35 (89.7)	5.87
Homosexual (#)	0 (0.0)	0 (0.0)	1 (2.6)	
Bisexual (#)	2 (5.0)	1 (2.9)	0 (0.0)	
Prefer not to say (#)	0 (0.0)	0 (0.0)	1 (2.6)	
Education level				
First year (#)	11 (27.5)	8 (22.6)	6 (15.4)	15.17
Second year (#)	7 (17.5)	6 (17.1)	15 (38.5)	
Third Year (#)	11 (27.5)	11 (31.4)	6 (15.4)	
Fourth year (#)	11 (27.5)	10 (28.6)	7 (17.9)	
Fifth year (#)	0 (0.0)	0 (0.0)	2 (5.2)	
Other (#)	0 (0.0)	0 (0.0)	1 (2.6)	

Values in parentheses are column percentages, except for age, which is presented as mean with standard deviation in parentheses. No significant differences between groups were observed.

Table 3 shows the results of 3 (experimental condition) x 2 (day/night 2 and day/night 3) repeated measures ANOVA assessment. There were no significant main or interaction effects observed across objective measures of TST_obj_, SE_obj_, SOL_obj_, WASO_obj_, or nocturnal heart rate variability. Additionally, there were no significant main or interaction effects among subjective variables including TST_sub_, SOL_sub_, WASO_sub_, the number of awakenings, or self-reported sleep quality conditions. However, there was a significant between-group difference in nocturnal heart rate, whereby participants in the control condition had significantly higher heart rate compared to those in the courteous ending condition, while those in the ghosting condition were not significantly different from those in the other two conditions. All observed effects were small in magnitude (|*d*|s < 0.20; partial η^2^s < 0.02).

**Table 3:** Comparison of sleep quality across study condition.

Sleep quality measures	Ghosting (*N* = 40)		Courteous ending (*N* = 35)		Control (*N* = 37)		F-statistic		
	Day 2	Day 3	Day 2	Day 3	Day 2	Day 3	Condition	Time	Interaction
Objective sleep quality									
Total sleep time (min)	419.41 (72.84)	424.99 (57.59)	405.13 (89.04)	420.29 (65.51)	407.14 (64.31)	411.15 (82.96)	0.55	0.92	0.16
Sleep efficiency (%)	89.53 (4.14)	89.53 (3.19)	90.57 (4.33)	90.43 (3.19)	89.56 (4.30)	90.03 (3.81)	0.94	0.11	0.26
Sleep onset latency (min)	15.50 (15.44)	14.80 (10.05)	14.59 (9.97)	14.44 (9.01)	15.84 (12.98)	16.64 (16.39)	0.26	0.00	0.10
Wake after sleep onset (min)	50.01 (25.74)	50.41 (16.82)	43.96 (25.24)	45.76 (17.56)	48.80 (24.50)	46.95 (23.52)	0.79	0.00	0.18
Heart rate (bpm)	61.72 (10.53)	62.90 (7.68)	62.00 (8.33)	61.51 (8.34)	66.37 (9.38)^a^	67.17 (8.73)^a^	3.97*	0.77	0.78
Heart rate variability (ms)	72.40 (46.82)	73.40 (44.28)	63.17 (37.92)	65.06 (35.91)	57.38 (28.96)	57.68 (27.64)	1.70	0.37	0.07
Subjective sleep quality									
Total sleep time (min)	436.53 (83.09)	440.10 (79.82)	436.71 (78.38)	429.74 (68.65)	417.22 (59.53)	432.78 (79.52)	0.45	0.23	0.58
Sleep onset latency (min)	21.20 (18.40)	20.69 (26.89)	20.11 (22.33)	18.20 (22.24)	15.76 (14.16)	14.65 (12.39)	0.04	0.33	0.04
Wake after sleep onset (min)	5.48 (11.08)	4.81 (9.05)	3.77 (6.21)	6.26 (12.92)	6.83 (19.03)	4.14 (9.05)	0.03	0.04	0.97
Awakenings (#)	1.50 (1.47)	1.16 (1.42)	1.09 (1.22)	1.46 (1.46)	1.42 (1.38)	1.21 (1.55)	0.03	0.14	1.88
Sleep quality (a.u.)	3.35 (0.83)	3.70 (0.82)	3.76 (0.70)	3.74 (0.71)	3.61 (0.90)	3.81 (1.04)	1.09	3.77	1.55

Sleep efficiency ranges from 0 to 100%; sleep quality is measured on a scale of 1 to 5 with higher scores indicating better sleep quality. Variables with different subscripts indicate significant differences; data with no subscripts illustrates no significant group differences. All values are mean (SD). BPM = beats/min; min = minute; ms = milliseconds; a.u. = arbitrary units.

*p < 0.05. ^a^*p* < 0.05 vs. courteous ending.

## Discussion

Given the results of this study, including small effect sizes, the hypotheses are not supported. This study illustrated that ghosting did not impact participants’ objective or subjective sleep quality. For the current study, some participants were ghosted by a research assistant posing as a potential partner, whereas others were softly let down by a research assistant posing as a potential partner, and others did not communicate with a research assistant during this study. Contrary to our hypothesis, we did not observe any significant influence of acute experimental ghosting on objective and subjective sleep parameters. Viewed from a methodological perspective, the present findings suggest that brief, low-investment ghosting manipulations may be insufficient to elicit strong physiological effects, thereby helping to define practical limits of experimental approaches to studying relational disengagement. This insight may inform future study designs that seek to model more ecologically valid or emotionally consequential forms of ghosting.

The results of this study reflect that for those initially connecting with a potential romantic partner, ghosting may not be a physiologically stressful experience. This finding contrasts with previous research indicating that ghosting can elevate heart rate and blood pressure (Langlais et al., 2024). The difference between these two studies could be related to demographic factors. In the current study, although the overall sample showed no effect on sleep quality, analyses within the female subsample alone did not reveal a significant effect either. Additionally, the current sample was larger and recruited from the southern central United States, whereas Langlais et al. (2024) included a smaller, all-female sample from the southeastern United States. Additionally, the ghosting manipulation was different between the two studies; the current study used text messaging and Langlais et al. (2024) used Snapchat. In the latter, read receipts are provided, which indicates when a message is opened and read; this information is not always provided through text messaging because of the device being used (i.e., a message sent from an Android device would not provide read receipts to an iPhone). We did not collect information about the device, which should be considered in future studies planning to use text messages. Additionally, the timing of conversations between participants and ‘potential’ partners was different. In Langlais et al. (2024), the conversations that lead to ghosting happened shortly after seeing the picture of an individual, whereas in the current study, the conversations happened in the evening. Also, in the current study, the effects of ghosting were tracked at different times. In Langlais et al. (2024), the consequences were tracked at the time that ghosting occurred, whereas the current study examined ghosting over the course of their sleep. It is possible that ghosting has strong acute physiological consequences and sharp declines in these consequences over time, particularly for relationships with low commitment and investment. This effect may reflect a tolerance for ambiguity, which may influence how participants experience and respond to ghosting, and contextual factors may further shape these reactions. Future research should consider tolerance for ambiguity to better understand the variability in responses to ghosting.

Beyond these methodological differences, these studies also focused on different physiological indicators. The current study focuses on objective and subjective sleep, whereas Langlais et al. (2024) focused on blood pressure and heart rate. Ghosting could impact certain physiological indicators, such as blood pressure, but not sleep. One potential explanation for the null findings is that ghosting may be less consequential for potential relationships that have not yet been established regarding sleep. When emotional investment is minimal and the relationship has not extended beyond 120 min of text messaging, the impact of ghosting for sleep quality may be diminished. Individuals may be more likely to rationalize or dismiss the experience, reducing its potential to disrupt sleep patterns compared to ghosting in more committed or emotionally invested relationships. When ghosting is used as a breakup strategy, ghosting may be more consequential for sleep. If ghosting occurs before a relationship forms, people may be less apt to lose sleep.

There are other explanations for why ghosting may not have had a significant impact on sleep quality. The participants never interacted with the ‘potential partner’ face-to-face, which makes it difficult to feel invested in the relationship. Studies have shown that face-to-face interactions are more meaningful in the context of romantic relationships (De Netto et al., 2021; Kim and Dindia, 2011; Ruppel et al., 2017). Additionally, the ambiguity of the relationship makes it hard for the ghosting experience to be consequential. The text message conversations could be seen as romantic, or could be perceived as platonic. Even though this study was under the guise of romantic matchmaking, some could have seen the text message experiment simply as friendly and attempting to get to know someone rather than seeking a potential romantic partner. Other external stressors cannot be ruled out as well. For college students, there are many competing stressors, such as extracurricular activities, jobs, peer and other close relationships, and grades (Arnett, 2000). These stressors may impact participants’ sleep quality more than ghosting experiences. External stressors should be controlled in future studies.

This rationale is also supported by the central tenets of belongingness theory (Baumeister and Leary, 1995). According to the theory, people have a fundamental need to form meaningful interpersonal connections, and being rejected can lead to emotional distress and other associated physiological consequences, including poor sleep. However, when individuals are ghosted via text by someone they have never met in person, the impact on their sense of belonging may be minimal due to limited emotional investment, lack of physical interaction, and the ambiguous or casual nature of the relationship. Moreover, the normalization of ghosting within dating culture and the tendency to cognitively distance oneself from shallow online connections may buffer the psychological effects (Koessler et al., 2019; LeFebvre et al., 2019; LeFebvre and Carmack, 2020). In such contexts, the ghosting experience may not rise to the level of a perceived relational loss sufficient to disrupt sleep quality, thereby attenuating the negative outcomes predicted by belongingness theory.

Although the present study did not detect statistically significant effects of experimental ghosting on either objective or subjective sleep outcomes, it is important to clarify the nature of these null findings. Across indicators, observed effects were uniformly small in magnitude and inconsistent in direction, suggesting an absence of meaningful change rather than suppressed or unstable effects. Conceptually, these results can be interpreted relative to a smallest effect size of interest, wherein only effects large enough to reflect practically meaningful disruptions to sleep would be theoretically consequential. The pattern observed here indicates that any effects of acute, early-stage ghosting on sleep (if present) are likely below such a threshold. While formal equivalence testing was not conducted, the convergence of near-zero effects across multiple sleep indicators strengthens confidence that the null findings reflect a true lack of substantive impact under the conditions studied. This framing aligns with recent recommendations to interpret null results not merely as nonsignificant, but as informative when effect sizes are small, consistent, and theoretically bounded.

One additional finding warrants cautious interpretation, which is that participants in the control condition exhibited slightly higher nocturnal heart rate relative to those in the courteous ending condition. This effect was not mirrored across other objective or subjective sleep indicators, nor was it part of a broader, theoretically coherent pattern, suggesting it should be interpreted conservatively. One possible explanation is that brief, polite social interaction may have exerted a mild regulatory or calming influence prior to sleep, consistent with work linking social connection to physiological downregulation. However, given the isolated nature of this effect, its small magnitude, and the absence of converging evidence across outcomes, it may also reflect a chance finding rather than a meaningful physiological consequence of the manipulation. Accordingly, this result should not be overinterpreted and instead underscores the importance of replication and more comprehensive physiological assessment in future experimental work on relational stress and sleep.

## Implications

The results of this study offer several important implications for both research and practice. First, the null findings challenge assumptions grounded in belongingness theory by suggesting that ghosting, when experienced in the context of flirting or a potential romantic relationship, may not be sufficiently impactful to disrupt sleep in the short term. This pattern suggests that not all instances of ghosting are equally distressing and that the prevalence of clinically or physiologically meaningful distress may be lower in low-investment or ambiguous relational contexts. In real-world dating environments, where brief and noncommittal interactions are common, ghosting may be experienced as disappointing or frustrating without necessarily producing acute disruptions to core regulatory processes such as sleep. This rationale should consider contextual factors, such as relationship depth and emotional investment, in moderating the psychological and physiological consequences of relational disruptions. Researchers should be cautious in assuming uniform negative outcomes of ghosting across all relationship stages and consider relationship context when interpreting its effects.

Second, the study demonstrates the feasibility of using a controlled, experimental protocol to simulate a ghosting experience and assess real-time physiological and subjective outcomes. This methodological contribution is significant, as it provides a foundation for more rigorous, ethically sound investigations of relational stressors within experimental settings. At the same time, because the present manipulation represents an early-stage and relatively low-investment interaction, it may be best understood as a lower-bound test of ghosting-related distress. Future studies may build on this design by modeling conditions that more closely resemble real-world situations associated with heightened distress, such as longer interaction histories, stronger expectations of continuity, or greater emotional investment. Future studies may also build upon this design by extending the monitoring period, incorporating measures of emotional reactivity, or examining long-term consequences of ghosting across different romantic relationship types (e.g., established relationships, friends-with-benefits, etc.).

Finally, the findings have implications for mental health practitioners and educators working with emerging adults. The absence of short-term sleep disruptions may suggest a degree of resilience or normalization of ghosting in the dating culture of young adults. Still, it is important to recognize that ghosting may have cumulative or delayed effects, particularly for individuals with pre-existing vulnerabilities. At a population level, this finding may help explain why ghosting is widely reported yet does not uniformly result in severe or immediate distress. However, this should not be interpreted as evidence that ghosting is harmless. Rather, distress may be conditional, cumulative, or delayed, particularly when ghosting occurs in more emotionally meaningful relationships or among individuals with greater vulnerability. Interventions that help young adults build emotional regulation skills and foster realistic expectations around modern dating dynamics could mitigate potential harm in more emotionally charged situations.

## Limitations and conclusions

While this study advances understanding on the phenomenon of ghosting, it is not without limitations. First, the study relied on a group of college students enrolled at the same university, which restricts generalizability. This sample was also relatively homogeneous, comprised of large amounts of women and white participants. Although the study was powered to detect effects in the medium range, no prior research has established expected effect sizes for the impact of ghosting on sleep quality. As a result, it is possible that the true effect is smaller than anticipated, in which case the current design may have lacked sufficient power to detect it. Future work should consider using power analyses based on small effect sizes to ensure adequate sensitivity, particularly as this area of research continues to develop and more precise estimates become available, and recruit larger, more diverse participants to provide stronger power to generalize to other populations. Second, the study asked participants to talk to a potential partner, and the degree to which they were seriously pursuing these potential partners likely varied. It is possible that some people may have considered these conversations friendly, others flirting, and others innocuous. Because this motivation is unclear, and the texting conversations were limited to 90–120 min, some participants may not have felt attached to the ‘potential partner’ who was texting them, likely explaining the lack of differences across the three conditions. We also did not analyze the number of messages exchanged or response latency, which could have provided an index of individual engagement in the conversation. Future research should include such measures to better capture variability in responses to ghosting. Another limitation is the validity in a two-hour texting conversation. Although long enough to engage in meaningful dialogue, not all young adult interactions naturally last 2 h; some may be shorter or more sporadic, so the study may overestimate engagement compared to typical texting behavior. Future studies should include an increase in manipulation checking to ensure that the ghosting experience is valid. More precisely, this study is limited in that it did not capture rejection, ambiguity, emotional impact, or perceived ghosting. Future studies should consider capturing perceptions of the ghosting experiences throughout the study, rather than only on the final day of the study, while also controlling for perceived ostracism, relational ambiguity, and felt rejection.

In addition, extended conversations could lead to fatigue, boredom, or reduced attention, which may influence responses or emotional reactions independent of the experimental manipulation. Even within a two-hour window, participants may differ widely in how frequently they respond, leading to variability in actual interaction time and message exchange, which could affect the consistency of the manipulation. It is important to add that the ethical review board did not approve conversations extending beyond 2 h to safeguard for participants. Although this ethical decision was made in the best interests of participants, the results of this study, which demonstrate primarily null effects of ghosting on sleep, can be used to justify longer periods of interaction in experimental designs regarding ghosting. Although ghosting often happens online (LeFebvre and Fan, 2020; Navarro et al., 2020), adding an in-person face-to-face component to this experiment could have increased validity and align more closely with tenets of belongingness theory. Future studies are encouraged to capture more realistic experiences of ghosting or use existing relationships to ghost (briefly and ethically) to determine the physiological consequences of this experience. Also, participants’ subjective assessments of the ghosting experience were not collected, which limits the ability to examine correlations between perceived intensity of ghosting and changes in sleep quality or physiological outcomes. Including such self-reports in future studies would allow for more nuanced analyses and help clarify individual differences in responses to ghosting.

Another limitation is the definition of ghosting. Although the manipulation met several core components of ghosting (being digitally enacted and involving a unilateral, unexplained cessation of communication), it may not have fully captured participants’ perceptions of ghosting as defined in prior work (Schokkenbroek et al., 2025). Specifically, we did not include manipulation checks throughout the experiment assessing whether participants experienced the interaction as a meaningful relationship or interpreted the sudden silence as an intentional termination rather than general rejection, temporary delay, or broader forms of ostracism, except on the last day of the study (D3). As a result, some participants may not have construed the experience as ghosting in the definitional sense, which may attenuate or obscure the effects under investigation. Future research would benefit from incorporating direct assessments of perceived relationship meaningfulness and the interpretation of communication cessation, or from using stronger behavioral cues, such as visible blocking features within an app, to more clearly signal relationship termination. These refinements would help ensure that experimental manipulations align more closely with participants’ lived experiences of ghosting. Also, the study did not assess individual factors such as message content, prior ghosting experiences, or participants’ subjective perceptions, which may have influenced responses. Future research should include these variables to better understand how ghosting impacts sleep and physiological outcomes. Finally, despite utilization of a randomized assignment, some between-group differences were observed, particularly in nocturnal HR. Specifically, nocturnal HR was slightly higher in the control condition compared to either experimental intervention group. There was no interaction effect of experimental condition and study session on nocturnal HR, suggesting that the between-group differences in HR were a spurious outcome associated with randomization. While differing levels of baseline HR between groups may impact reactivity to each experimental condition, the lack of notable HR responsiveness across experimental conditions suggests that group HR differences did not impact the present findings.

Despite these limitations, this study builds on previous research regarding ghosting in romantic relationships by using subjective and objective markers of sleep with participants who were communicating with someone who they believed matched with them romantically. Future studies are encouraged to extend this research by examining communication for longer periods of time with potential romantic partners. Through this study and future research, more information can be disseminated that can support interpersonal health as a result of relationship experiences.

## Data Availability

The raw data supporting the conclusions of this article will be made available by the authors, without undue reservation.
